# Mulberry and *Hippophae*-based solid beverage promotes weight loss in rats by antagonizing white adipose tissue PPARγ and FGFR1 signaling

**DOI:** 10.3389/fendo.2024.1344262

**Published:** 2024-03-15

**Authors:** Xiao-Ting Zhou, An-Qi Zhu, Xiao-Min Li, Ling-Yue Sun, Jian-Gang Yan, Nin Luo, Shi-Sheng Chen, Zebo Huang, Xin-Liang Mao, Kun-Ping Li

**Affiliations:** ^1^ Key Laboratory of Glucolipid Metabolic Disorders, Ministry of Education of China; Institute of Chinese Medicinal Sciences, Guangdong Pharmaceutical University, Guangzhou, China; ^2^ Research & Development Division, Perfect Life & Health Institute, Zhongshan, China; ^3^ Research & Development Division, Perfect (Guangdong) Co., Ltd., Zhongshan, China; ^4^ School of Food Science and Engineering, South China University of Technology, Guangzhou, China

**Keywords:** obesity, GSEA, PPARγ, metabolomics, transcriptomics, weight-loss

## Abstract

Obesity, a multifactorial disease with many complications, has become a global epidemic. Weight management, including dietary supplementation, has been confirmed to provide relevant health benefits. However, experimental evidence and mechanistic elucidation of dietary supplements in this regard are limited. Here, the weight loss efficacy of MHP, a commercial solid beverage consisting of mulberry leaf aqueous extract and *Hippophae* protein peptides, was evaluated in a high-fat high-fructose (HFF) diet-induced rat model of obesity. Body component analysis and histopathologic examination confirmed that MHP was effective to facilitate weight loss and adiposity decrease. Pathway enrichment analysis with differential metabolites generated by serum metabolomic profiling suggests that PPAR signal pathway was significantly altered when the rats were challenged by HFF diet but it was rectified after MHP intervention. RNA-Seq based transcriptome data also indicates that MHP intervention rectified the alterations of white adipose tissue mRNA expressions in HFF-induced obese rats. Integrated omics reveals that the efficacy of MHP against obesogenic adipogenesis was potentially associated with its regulation of PPARγ and FGFR1 signaling pathway. Collectively, our findings suggest that MHP could improve obesity, providing an insight into the use of MHP in body weight management.

## Graphical Abstract

Efficacy of MHP against diet-induced obesity is validated in rats. Integrated metabolomics and transcriptomics indicate that MHP is able to facilitate weight loss through regulating PPARγ pathway and redressing adipose tissue dysfunction.

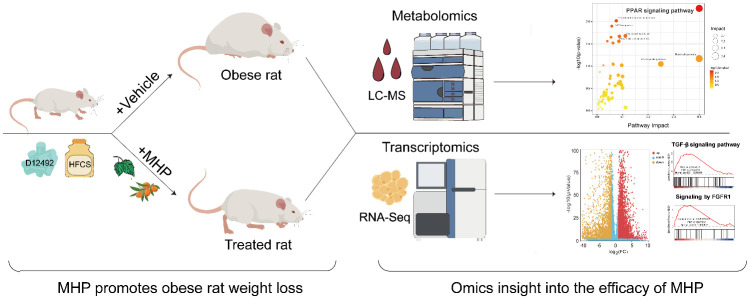

## Highlights

- MHP promotes weight and adiposity loss of diet-induced obese rats.
- Metabolomics shows MHP efficacy is closely related to PPAR signaling pathway.
- Transcriptomics indicates MHP rectifies white adipose tissue dysfunction.
- Efficacy of MHP is associated with regulation of PPARγ and FGFR1 signaling.

## 1 Introduction

Overweight or obesity is characterized by an excessive accumulation of adipose tissue and has become a global epidemic (1, 2). Obesity is highly associated with the development of hyperlipidemia, nonalcoholic fatty liver disease (NAFLD), type 2 diabetes mellitus (T2DM), cardiovascular diseases and some types of cancer (3), posing one of the greatest threats to health. On the other hand, weight management has been shown to provide health benefits. For example, a moderate 5% weight loss has considerable health benefits, including decreased intra-abdominal and intra-hepatic fat and increased multiorgan insulin sensitivity and β cell function (4). Long-term intentional weight-loss also improves the morbidity, mortality, quality of life, and health-care cost (5, 6). Obesity guidelines have recommended weight loss of 5-10% as the goal for medically supervised weight loss (7, 8).

Given the great benefits of weight loss, a number of measures have been applied to realize the goal of 5% or more weight loss and weight maintenance in the past few decades, including active lifestyle modifications and pharmacological and surgical interventions. Among them, noninvasive drugs and dietary supplements have been regarded as promising approaches to address the deficit of lifestyle intervention and reduce the severity of bariatric surgery (9, 10). In the case of dietary supplements, usually no specific guidance from a professional doctor is required and often no strict restrictions are applied – they can be sold as functional beverage or health food (11). Thus, many people turn to weight loss-promoting dietary supplements in the hope to help them achieve their weight-loss goals. However, despite extensive investigations on the role of dietary bioactive substances with weight-loss potentials (12, 13), evidence to support the efficacy of dietary supplements *perse* for weight loss is limited (14). According to the U.S. FDA regulations, all dietary supplements must be safe, but unlike drugs, there are currently no efficacy requirements (13). Consequently, commercially available dietary supplements that are labeled for use in weight loss are multifarious, varying greatly in their efficacy (14, 15). Therefore, there is a pressing need to investigate the claimed efficacy of weight-loss dietary supplements as well as the underlying regulatory mechanisms in metabolic pathways.

In our recent screening tests, a number of dietary supplements are found to have weight-loss activities. Among them, MHP, a solid beverage which is mainly consisted of mulberry leaf aqueous extract and *Hippophae* protein peptides as well as L-arabinose, showed potential protective effects against high-fat diet-induced obesity and hyperlipidemia. Mulberry leaf has been traditionally used to lower blood glucose and lipids, which is supported by their high content of flavonoids and alkaloids (16). *Hippophae rhamnoides* L. has also been shown to have weight-loss and lipid-lowering activities (17, 18). *Hippophae* protein peptides prepared from *H. rhamnoides* seeds have also been proved effective in combating memory impairment (19) and also shown to have hypoglycemic and anti-inflammatory activities (20). These findings suggest that MHP has weight-loss potential, but its efficacy and underlying mechanisms have not been fully investigated.

Variation of endogenous metabolites reflects the alterations of global metabolic homeostasis in cells and organisms, which can be investigated by metabolomics. As a robust analytical tool, metabolomics has been increasingly used in the studies of diagnostic biomarkers, fundamental pathogenic mechanisms, and therapeutic targets (21). By integrating with transcriptomics data and using such as Ingenuity Pathways Analysis, MetaCore and Reactome software, algorithmically constructed metabolic networks can be generated to provide more insight than phenotypes and biochemical results analyzed individually (22).

In this study, we first evaluated the effects of MHP on a high-fat high-fructose (HFF) diet-induced rat model of obesity and then explored the underlying mechanisms by integrated metabolomics and transcriptomics analysis. Our results showed that MHP rectified white adipose tissue dysfunction and had weight-loss activity, which was related to its regulation on PPARγ (peroxisome proliferator-activated receptor gamma) and FGFR1 (fibroblast growth factor receptor 1) signaling.

## 2 Materials and methods

### 2.1 Chemicals and reagents

Liquid chromatography-tandem mass spectrometry (LC-MS) grade methanol (MeOH) and acetonitrile (ACN) were purchased from Fisher Scientific (Loughborough, UK). Formic acid was obtained from TCI (Shanghai, China). 2-Amino-3-(2-chlorophenyl) propionic acid (internal standard) and ammonium formate were obtained from Sigma Aldrich (Shanghai, China). Hematoxylin plus Eosin dye kit (DH0006) was purchased from Leagene (Beijing, China). Ultrapure water was generated using a Milli Q system (Millipore, Bedford, USA).

### 2.2 MHP preparation and analysis

MHP is mainly consisted of mulberry leaf aqueous extract, *Hippophae* protein peptide and L-arabinose. In the present study, MHP (batch No. 20220623) was manufactured in a Good Manufacturing Practice (GMP) pilot plant following specific quality assurance instructions for the nutritional ingredients, heavy metals and microorganisms (see **Supplementary Table S1** in **Supplementary Materials**).

### 2.3 Animals experiment

Male Sprague-Dawley rats (5-6 weeks old) were supplied by Guangdong Medical Laboratory Animal Center (Guangzhou, China). Animal study protocols were approved by the Institutional Animal Care and Use Committee of the Guangdong Pharmaceutical University. All animal studies were conducted in accordance with the ARRIVE (Animal research: reporting of *in vivo* experiments) guidelines (23). All animals were maintained under specific pathogen-free conditions, housed with 12 h light/dark cycles at controlled 22-24°C and 60-65% humidity. After acclimatization on a normal chow diet for 7 days, the rats were randomly divided into control group and high calorie diet group. As previously described (22, 24), the control group was fed with standard chow and the high calorie diet group was fed with a high-fat diet plus HFCS-sweetened drink. Two weeks later, an elimination about 20% of obesity-resistant rats was performed and the calorie diet group was divided into HFF group, MHP-H and MHP-L group (n=8 for each group). Except the rats of normal group, all the other rats were fed with a high-fat high-fructose (HFF) diet, and the rats of MHP-H and -L group were supplemented with MHP at a dose of 5.25 g/kg/day and 0.875 g/kg/day by gavage respectively.

The intervention lasted for 8 weeks. Body weight of each rat was measured twice per week throughout the experiment. In addition, food and drink intake were monitored, and the energy intake and energy efficiency (body weight gain(g)/total energy intake (kcal)) of each group were calculated.

### 2.4 Sample collection

At the end of the experiment, all animals were anaesthetized with pentobarbital sodium after an overnight (12h) fast. Blood and tissues were harvested as previously described (25). Blood was collected for serum metabolomics analysis. The inguinal white adipose tissue (iWAT) was also collected and weighted. Part of iWAT was fixed with 4% PFA for histological examination, while the rest was snap frozen in liquid nitrogen. Samples were frozen at -80°C until analyzed for metabolomics (plasma) or transcriptomics (iWAT tissue).

### 2.5 Body composition analysis

Body composition analysis was carried out as previously described using an NMR analyzer Minispec LF90II body composition analyzer (Bruker Optics, Billerica, MA, USA) 1 day prior to sacrifice (25). The adiposity, the ratio of lean mass to body weight, and the ratio of fluid mass to body weight were calculated.

### 2.6 Metabolomics analysis

The untargeted metabolomics analysis was performed as described (26). All the samples were thawed at 4°C. To 100 µL of serum, 400 µL of methanol was added and vortexed for 1 min. The mixture was then centrifuged for 10 min at 12,000 rpm and 4°C, and the supernatant was transferred, concentrated and dried in vacuum. Next, the dry samples were redissolved with 150 µL of 80% methanol solution containing 2-chlorolphenylalanine (4 ppm) and filtered through 0.22 μm membrane prior to LC-MS detection. A small aliquot of each sample was pooled together as the quality control sample.

The LC analysis was performed on a Vanquish UHPLC System (Thermo Fisher Scientific, USA) using an ACQUITY UPLC^®^ HSS T3 (2.1×100 mm, 1.8 µm) column (Waters, Milford, USA). The column was maintained at 40 °C. The flow rate and injection volume were set at 0.3 mL/min and 2 μL, respectively. For LC-ESI (+)-MS analysis, acetonitrile containing 0.1% formic acid (B1) and water containing 0.1% formic acid (A1) were used as mobile phase, while for LC-ESI (–)-MS analysis, pure acetonitrile (B2) and 5 mM ammonium formate (A2) were used. Chromatographic separation was carried out under the following gradient: 0~1 min, 8% B1(B2); 1~8 min, 8%~98% B1(B2); 8~10 min, 98% B1(B2); 10~10.1 min, 98%~8% B1(B2); and 10.1~12 min, 8% B1(B2). Simultaneous MS and data-dependent MS/MS acquisition of metabolites mass spectrometry data were performed on Orbitrap Exploris120 with ESI ion source (Thermo Fisher Scientific, USA). The sheath gas pressure and auxiliary gas were 40 and 10 arb, respectively. The spray voltage was 3.50 kV and -2.50 kV for ESI (+) and ESI(-), respectively. The capillary temperature was 325 °C. MS^1^ scan range of m/z was from 100 to1000 and MS^1^ resolving power was set at 60000 FWHM. The number of data dependent scans per cycle was set at 4. The MS/MS resolving power was set at 15000 FWHM. The normalized collision energy was 30 eV.

The raw data were first converted to mzXML format by MSConvert in ProteoWizard software package (v3.0.8789) and processed using R XCMS (v3.12.0) for feature detection, retention time correction and alignment (27, 28). The metabolites were identified by accuracy mass and MS/MS data which were matched with HMDB (http://www.hmdb.ca), KEGG (https://www.genome.jp/kegg/), mzcloud (https://www.mzcloud.org) and the in-house metabolites database of Panomix Biomedical Tech Co., Ltd. (Shuzhou, China).

### 2.7 Metabolic pathway and function analysis

The metabolic pathway and function enrichment analysis was proceeded as in previous studies (25, 29). PCA (principal component analysis) and OPLS-DA (orthogonal partial least squares discriminant analysis) were applied to discriminate the groups by R ropls (v1.22.0) package (30). In order to screen the differential metabolites (DMs), *p* value < 0.05 and VIP (variable projection importance) value > 1 were set by fault as the threshold. Using the generated DMs, pathway enrichment analysis was performed using the online metabolomics tool of Biodeep Platform (http://www.biodeep.cn/) to visualize and interpretate the data.

### 2.8 RNA-Seq

RNA-Seq was performed as reported previously (22). Briefly, RNA from the iWAT was extracted using Trizol reagent kit (Invitrogen, USA) following the manufacturer’s instruction. The cDNA library was constructed and sequenced on the Illumina (Novaseq 6000) by Gene Denovo Biotechnology Co. (Guangzhou, China). After the raw data were filtered, the clean reads were used for identification the differentially expressed genes (DEGs) by DESeq2 package. The level of mRNA FPKM (fragments per kilobase of exon model per million fragments mapped reads) value was also calculated. Genes with *p* value < 0.05 and |Fold change|> 1.5 were categorized as DEGs, and then used for gene ontology (GO) function analysis and Kyoto Encyclopedia of Genes and Genomes (KEGG) pathway analysis. Gene Set Enrichment Analysis (GSEA) was implemented on the Java GSEA platform while gene sets with NES absolute value >1, *p* value <0.05 and FDR value < 0.25 were considered statistically significant. All the raw data generated by RNA-Seq were submitted to the GenBank’s Sequence Read Archive (https://www.ncbi.nlm.nih.gov/sra/PRJNA1063764).

### 2.9 Statistical analysis

All data are expressed as means ± standard deviation (SD). A Student’s *t*-test or one-way analysis of variance (ANOVA) was carried out. *p* value < 0.05 was considered statistically significant. R (https://www.rproject.org) and Graphpad Prism 6.0 software (GraphPad, CA, USA) were used for graphics.

## 3 Results and discussion

### 3.1 MHP facilitates weight loss of HFF-diet fed obese rats

**Figure 1:**
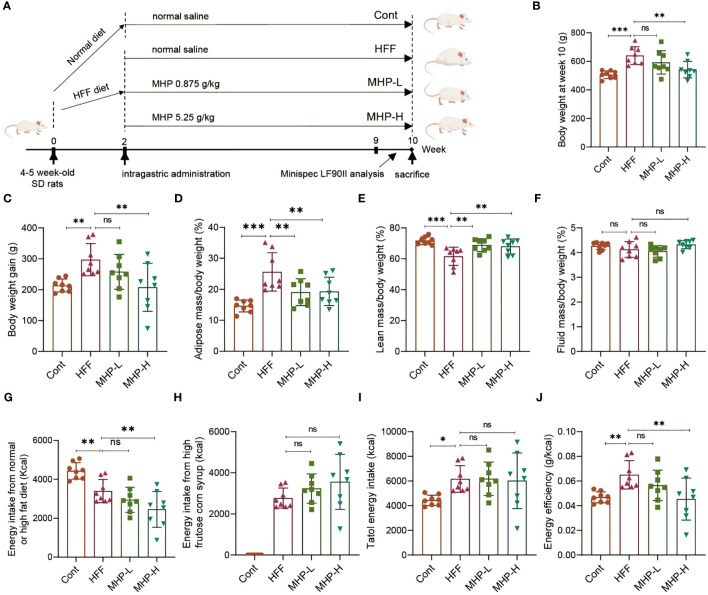
MHP facilitates weight loss in a high-fat high-fructose diet-induced obese rats. **(A)** SD rats were fed either a normal chow diet or a high-fat diet plus HFCS-sweetened drink (HFF diet). Two weeks later, an elimination about 20% of obesity-resistant rats from the HFF diet fed group was performed and the rest rats were divided into HFF group, MHP-H group and MHP-L group (n=8 for each group). The control and HFF group of rats received deionized water (vehicle), while the MHP-H and MHP-L groups were supplemented with MHP at a dose of 5.25 g/kg/day and 0.875 g/kg/day, respectively, via oral gavage until the end of the experiment. **(B, C)** Body weight and body weight gain. **(D–F)** Adiposity, lean and fluid index. **(G, H)** Energy intake from normal or high-fat diet and high-fructose corn syrup sweetened drink, respectively. **(I)** Total energy intake. **(J)** Energy efficiency of each group of rats. Cont, control group. HFF, high-fat high-fructose diet fed group. MHP-L and MHP-H are low dose- and high dose-MHP supplemented groups, respectively. MHP, a mulberry and *Hippophae*-based solid beverage. Data are expressed as mean ± SD (n =8). *p < 0.05, **p < 0.01, ***p < 0.001 vs. HFF group.

In order to address the efficacy of MHP for weight loss, a serial of experiments were performed on a HFF-induced rat model of obesity (**Figure 1A**). As shown in **Figures 1B, C**, the body weight of the HFF group rats was significantly increased compared with normal rats, but 8-week intervention with a high dose of MHP ameliorated the body weight gain caused by HFF diet.

Since obesity is associated with excessive fat accumulation, the lean mass and fluid mass of each group rats were also measured. As expected, a high dose of MHP treatment significantly decreased the adiposity and increased the lean index, while there was no difference in the ratio of fluid to body weight (**Figures 1D–F**). In order to investigate whether the increase of body weight or WAT in rats was related to food, drink or total energy intake, we measured the calory intake and calculated the energy efficiency. It was found that there was no difference in total energy intake between the HFF group and MHP group, suggesting that the weight loss was not due to a reduced total energy intake and that MHP did not inhibit the rat appetite (**Figures 1G–J**).

### 3.2 Multivariate statistical analysis of metabolomics data reveals HFF-diet-stressed metabolic alterations

**Figure 2:**
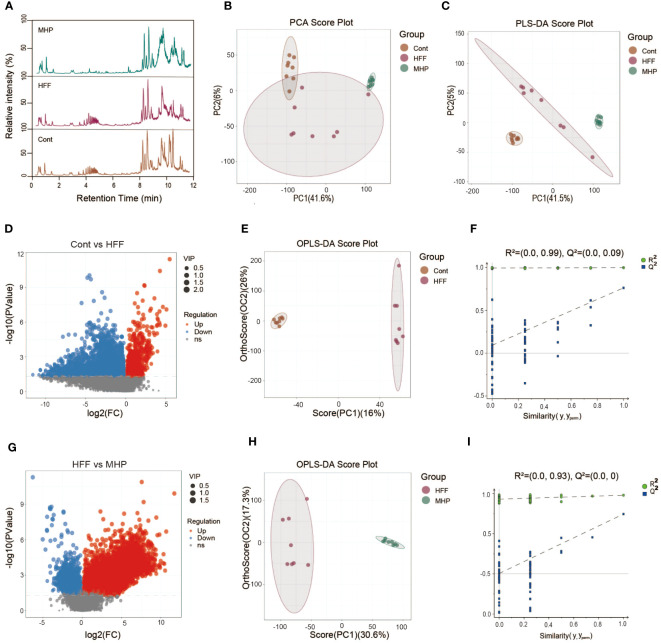
Multivariate statistical analysis of the LC-MS data acquired under positive ion mode. **(A)** Representative ESI(+)-MS total ion chromatograms of three groups of serum samples; **(B, C)** Principal component analysis (PCA) and partial least squares-discriminate analysis (PLS-DA) score plots; **(D, G)** Volcano plot of MS^1^ significant differential metabolites; **(E, H)** Pair-wise orthogonal projections to latent structures discriminant (OPLS-DA) scores plot; **(F, I)** Statistical validation of the OPLS-DA model by permutation testing.

The metabolic profiles of animals are dynamically changing, which can reflect the physiologic or pathologic conditions. Although it is complicated, metabolomics analysis can present some useful hints for mechanistic investigation (31). To study the underlying mechanisms for MHP efficacy, we performed a LC-MS based metabolomic profiling of serum collected from all the three groups of rats. Considering their efficacy, the MHP-H group was chosen for metabolomics analysis. Representative total ion chromatograms of LC-MS profiling of Cont, HFF and MHP groups of rats under positive ion mode are shown in **Figure 2A**. Mass spectrometry-based metabolomics studies require quality control to obtain reliable and high-quality metabolomics data (32). For this, the PCA score plots were used for both positive and negative modes (**Supplementary Figures S1A, B**). The PCA (R^2^X = 0.525) and PLS-DA score plots (R^2^X =0.505, R^2^Y =0.999, and Q^2 =^ 0.936) generated by multivariate statistical analysis based on acquired data indicated that the metabolome of the three groups of rats were distinct from each other (**Figures 2B, C**).

The volcano plot of the metabolites represented by MS data also showed obvious difference between the control and HFF group (**Figure 2D**), so do the HFF and MHP groups (**Figure 2G**). In order to maximize the discrimination between every two groups and screen out the differential metabolites, OPLS-DA analysis was carried out. As a result, in the OPLS-DA score plot of Cont versus HFF group, clear difference was presented while the cumulative R^2^X, R^2^Y, Q^2^ value was 0.488, 0.998 and 0.762, respectively (**Figure 2E**). Similarly, HFF group was also different from MHP group in the OPLS-DA score plot with the cumulative R^2^X, R^2^Y, Q^2^ values being 0.479, 0.98, and 0.745 respectively (**Figure 2H**). R^2^X and R^2^Y are known to be the cumulative model variation in X and Y, while Q^2^ is the cumulative predicted variation. In general, the values of these parameters that are approaching 1.0 indicate that a model is stable and has predictive reliability. A permutation test with 200 iterations confirmed that the constructed OPLS-DA model was valid and not over-fitted, as the original R^2^ and Q^2^ values to the right were significantly higher than the corresponding permutated values to the left (33). The value of R^2^ was 0.99 and Q^2^ was 0.09 in Cont versus HFF group (**Figure 2F**), while R^2^ was 0.93 and Q^2^ was 0.0 between HFF and MHP group (**Figure 2I**).

**Figure 3:**
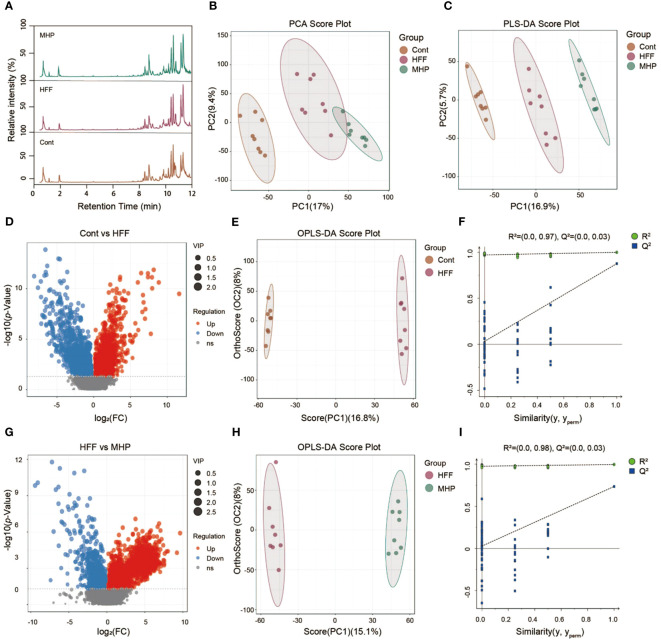
Multivariate statistical analysis of LC-MS data acquired under negative ion mode. **(A)** Representative ESI(+)-MS total ion chromatogram of serum samples; **(B, C)** Principal component analysis (PCA) and Partial least squares-discriminate analysis (PLS-DA) score plots; **(D, G)** Volcano plots of MS^1^ significant differential metabolites; **(E, H)** Pair-wise orthogonal projections to latent structures discriminant (OPLS-DA) scores plots; **(F, I)** Statistical validation of the OPLS-DA model by permutation testing.

Similarly, the LC-MS data under negative ion mode of metabolic profiles were acquired and analyzed. Representative total ion chromatograms of Cont, HFF and MHP groups were shown in **Figure 3A**. The PCA (R^2^X =0.533) and PLS-DA (R^2^X =0.226, R^2^Y =0.995 and Q^2 =^ 0.914) score plots showed clear differences among the Cont, HFF and MHP groups (**Figures 3B, C**). The volcano plots originated from ESI(-)-MS data also showed distinct difference between Cont and HFF groups (**Figure 3D**) as well as between HFF and MHP groups (**Figure 3G**). Following the aforementioned procedure, the OPLS-DA score plot of Cont versus HFF group also showed clear difference with the cumulative R^2^X at 0.248, R^2^Y at 0.999, and Q2 at 0.876 (**Figure 3E**). The cumulative R^2^X, R^2^Y and Q^2^ value were 0.231, 0.996, and 0.735 respectively between HFF and MHP groups (**Figure 3H**). Moreover, a permutation test confirmed that the present OPLS-DA model was valid and not over-fitted. The detailed parameters were as the following: R^2^ was 0.97 and Q^2^ was 0.03 in Cont versus HFF group (**Figure 3F**), while R^2^ was 0.98 and Q^2^ was 0.03 between HFF and MHP groups (**Figure 3I**).

### 3.3 MHP efficacy is associated with PPAR signaling pathway as revealed by metabolomics

**Figure 4:**
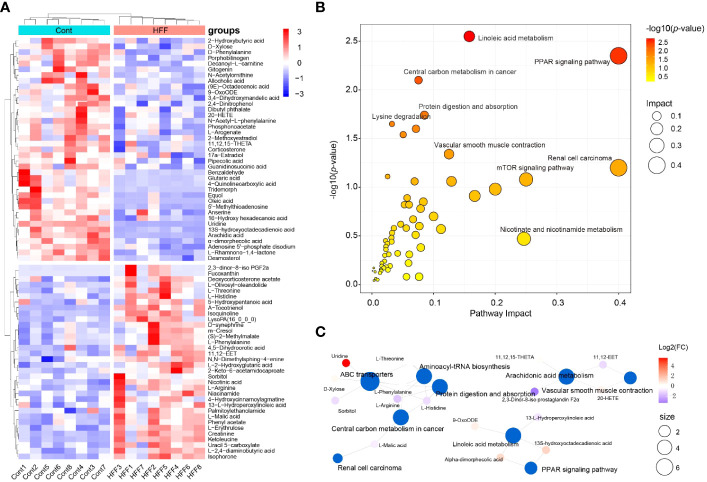
Rat serum metabolome is significantly altered when challenged by HFF diet. Untargeted metabolic analysis was performed through Vanquish UHPLC tandem Orbitrap Exploris120 MS system. The compounds were identified and quantitated by accuracy mass and MS/MS data which were matched with HMDB (http://www.hmdb.ca), KEGG (https://www.genome.jp/kegg/), mzCloud (https://www.mzcloud.org) and the in-house metabolites database of Panomix Biomedical Tech Co., Ltd. (Shuzhou, China). **(A)** Relative levels of 73 differential metabolites shown in heatmap. **(B)** Pathway enrichment analysis of the above 73 differential metabolites was performed using the online metabolomics tool of Biodeep Platform (http://www.biodeep.cn/) to visualize the results. **(C)** Metabolic network generated with differential metabolites during HFF versus control. Heatmap was generated by R pheatmap package using the 73 differential metabolites presented by metabolomic profiling of the rat serum samples of control and HFF group. HFF, high-fat diet and high-fructose corn syrup drink.

Metabolome reflects the holistic condition of an organism, presenting a global manifestation by a list of metabolites which are inextricably linked in terms of origination and metabolic pathways involved. Taking VIP > 1 and *p* < 0.05 as threshold values, a total of 73 serum small molecules were identified as the DMs between the control and HFF group rats when the ESI(+) and ESI(-) data were pooled together (**Supplementary Table S2** in **Supplementary Materials**). As shown in **Figure 4A**, the DMs clustered into two distinct groups in the heatmap, which showed that the serum metabolome was greatly altered after HFF diet consumption.

Individually, each metabolite involves in specific biochemical pathway and plays an important role in the delicate life process. For example, when compared with Cont group, the contents of oleic acid and uridine in serum of rats were considerably upregulated (fold change, FC >3.0) while α-tocotrienol was significantly downregulated (FC <0.5) in HFF group. In fact, oleic acid can induce oxidative damage and steatosis in hepatocytes both *in vitro* and *in vivo* (34). Uridine is one metabolite positively associated with obesity, and is also a potential biomarker of insulin resistance (35, 36). Tocotrienols possess powerful antioxidant, anticancer, and cholesterol-lowering properties (37), and tocotrienols supplementation not only inhibits body weight gain but also attenuates oxidation stress in HFD-treated mice (38). On the whole, however, the significantly changed DMs should be taking into account for a living organism. To this end, Cytoscape, MetaboAnalyst, MetaCore and other programs can generate algorithmic networks which can present more insights than the blunt results analyzed individually (25, 39). Thus, in order to illustrate and visualize the underlying metabolic stress induced by HFF diet, the above DMs were further analyzed using MetaboAnalyst 5.0 (www.metaboanalyst.ca) to interpret the apparently independent changes into altered canonical pathways and metabolic regulation networks. As shown in **Figure 4B**, pathway enrichment results suggest that the changes of metabolic profile in rats were related principally to (1) PPAR (peroxisome proliferator-activated receptors) signaling pathway, (2) linoleic acid metabolism, (3) renal cell carcinoma, and (4) other pathways. The metabolic network generated with DMs is visualized by Cytoscape and shown as **Figure 4C**. PPARs, including PPARα, PPARγ and PPARβ/δ, are fatty acid-activated transcription factors of the nuclear hormone receptor superfamily that regulate energy metabolism. PPAR signaling pathway is critical to coordinate many cellular events during normal and stress conditions (40).

**Figure 5:**
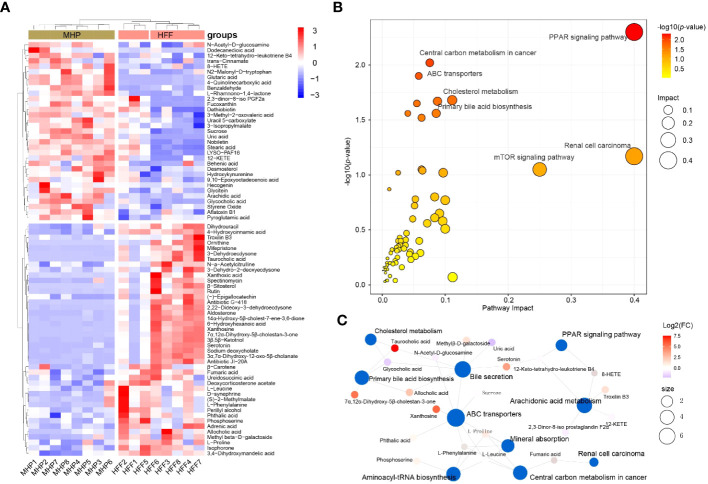
Metabolic remodeling in obese rat after a high-dose MHP supplementation. Untargeted metabolic analysis was performed using Vanquish UHPLC tandem Orbitrap Exploris120 MS system. The compounds were identified and quantitated by accuracy mass and MS/MS data which were matched with HMDB (http://www.hmdb.ca), KEGG (https://www.genome.jp/kegg/), mzCloud (https://www.mzcloud.org) and the in-house metabolites database of Panomix Biomedical Tech Co., Ltd. (Shuzhou, China). **(A)** Relative levels of 76 differential metabolites shown in heatmap. **(B)** Pathway enrichment analysis of the above 76 differential metabolites was performed using the online metabolomics tool of Biodeep Platform (http://www.biodeep.cn/) to visualize the results. **(C)** Metabolic network generated with differential metabolites during HFF versus MHP. Heatmap was generated by R pheatmap package using the 76 differential metabolites presented by metabolomic profiling of the rat serum samples of HFF and MHP group. HFF, high-fat diet and high-fructose corn syrup drink.

Likewise, using VIP > 1 and *p* < 0.05 as the thresholds, OPLS-DA generated 76 serum DMs between the HFF challenged rats and MHP supplemented rats based on the integrated ESI(+) and ESI(-) data (**Supplementary Table S3** in **Supplementary Materials**). The DMs can also be clustered into two distinct groups in the heatmap (**Figure 5A**), which demonstrates that MHP intervention can ameliorate the metabolism stress induced by HFF consumption. Among them, some individual metabolites are interesting. For instance, compared with HFF group, 6-hydroxyhexanoic acid was remarkably upregulated in MHP group (FC>3.0), which can significantly reduce high-fat diet induced weight gain and improve glucose intolerance and insulin resistance in mice (41). While the content of uric acid was significantly downregulated in MHP group (FC <0.5), and recent studies have confirmed that high level of serum uric acid is associated with obesity and metabolic syndrome (42). However, an algorithmically constructed metabolic network presents more insight than a serial of the individual metabolites (25). Therefore, the above DMs were also used to perform pathway enrichment analysis by MetaboAnalyst 5.0. As shown in **Figure 5B**, the changes of metabolic profile in rats can be related principally to (1) PPAR signaling, (2) renal cell carcinoma, (3) central carbon metabolism in cancer, and (4) other pathways. The metabolic network generated with DMs between HFF and MHP groups was shown as **Figure 5C**. Unexpectedly, in both **Figures 4B** and **5B**, PPAR signaling pathway seems to be the most impacted pathway, suggesting that this pathway is critical to coordinate the whole metabolism. Although more evidence may be needed, these results suggest that the efficacy of MHP is closely related to its regulation on the PPAR signaling pathway, which is worthy of further investigations.

### 3.4 MHP mitigates white adipose tissue transcriptome stress response in HFF-diet induced obese rats

**Figure 6:**
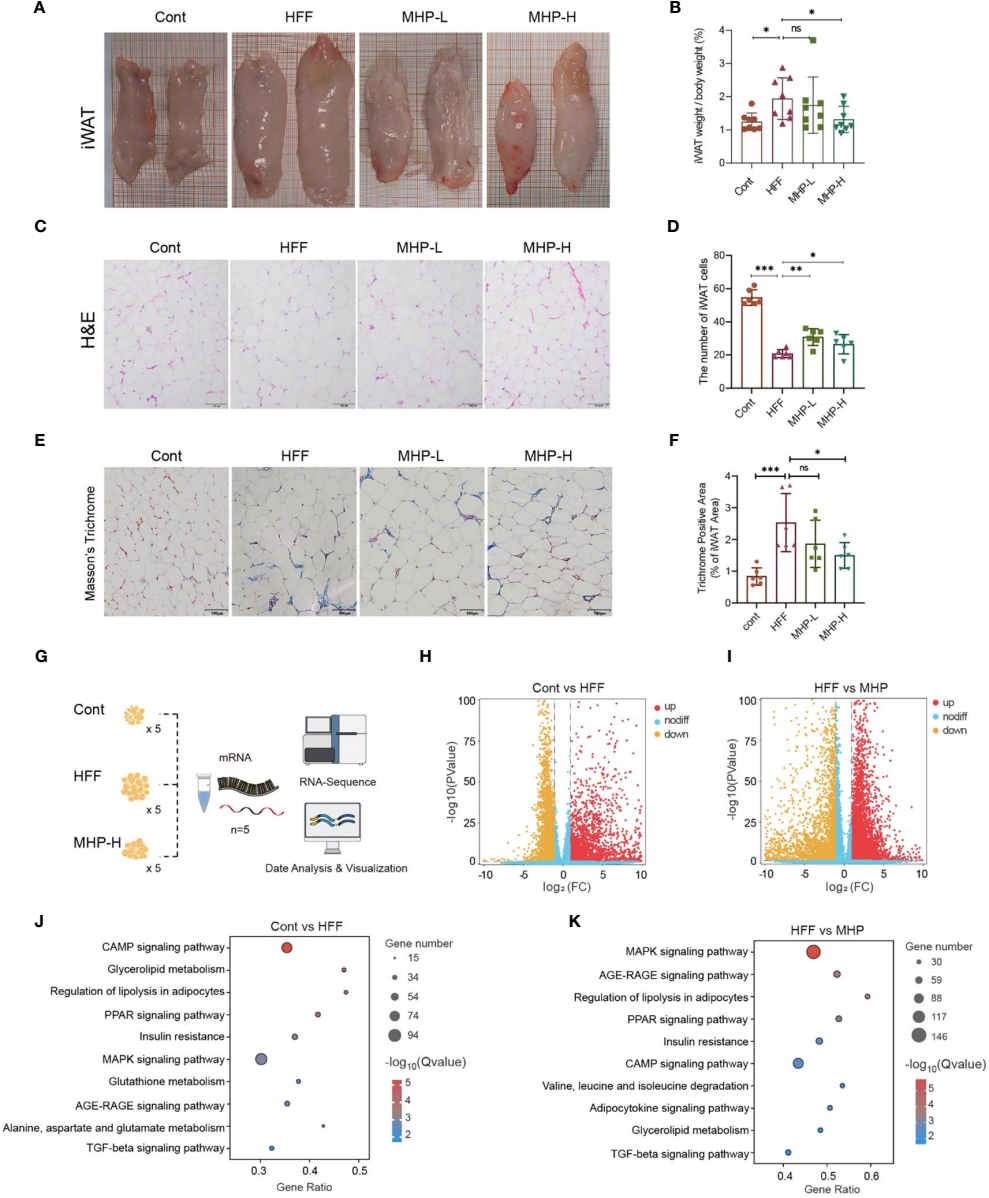
MHP improved white adipose tissue transcriptomic stress in HFF-induced obese rats. **(A, B)** Enlarged iWAT and increase in iWAT weight/body weight; **(C, D)** H&E stained iWAT sections and quantitative results of adipocytes. **(E, F)** Masson’s trichrome stained iWAT sections and quantitative analysis of collagenous fibrosis. **(G)** RNA-Seq analysis of iWAT; **(H, I)** Volcano plot of genes; **(J, K)** KEGG pathway enrichment analysis of DEGs. Cont, control group. HFF, high-fat high-fructose diet fed group. MHP-L and MHP-H are low dose- and high dose-MHP supplemented groups, respectively. MHP, a mulberry and *Hippo*phae-based solid beverage. GSEA, gene set enrichment analysis. iWAT, inguinal white adipose tissue. The access number of RNA-Seq raw reads is PRJNA1063764. (https://www.ncbi.nlm.nih.gov/sra/PRJNA1063764). Data are expressed as mean ± SD (for iWAT weight/body weight, n =8; for H&E stained and Masson’s trichrome stained sections, n=6). *p < 0.05, ***p < 0.001 vs. HFF group.

Abnormal WAT is a phenotypic indicator of over-weight or obesity. In the present study, HFF diet intake led to an enlarged iWAT (**Figure 6A**) and an increase in the ratio of iWAT weight to body weight (**Figure 6B**). Therefore, we expect that MHP supplementation may attenuate excessive lipid deposition. Indeed, pathohistological examination of iWAT sections, both with H&E staining and Masson’s trichrome staining, showed that the rats in MHP-H group had less hypertrophic adipocytes as compared with HFF group (**Figures 6C, D**) and collagenous fibrosis (**Figures 6E, F**). These results confirmed the efficacy of MHP on weight loss.

As metabolome changes should originate from its upstream effects, including proteome and transcriptome changes, thus we performed RNA-Seq analysis of iWAT to verify the influence of HFF on the transcriptomic profiles in white adipose tissue (**Figure 6G**). As shown in **Figure 6H, I**, an obvious difference was found between the control and HFF groups as well as between the HFF and MHP-H groups. By setting *p* < 0.05 and |Fold change| > 2.0 as thresholds, a total of 5095 DEGs and 7907 DEGs were screened out between control and HFF groups and between HFF and MHP groups, respectively (see **Supplementary Figure S2** in **Supplementary Materials**). Furthermore, KEGG pathway enrichment analysis of these DEGs showed that several pathways, including CAMP signaling, glycolipid metabolism, regulation of lipolysis in adipocytes, PPAR signaling, insulin resistance and MAPK signaling, were significantly changed by HFF diet (**Figure 6J**), confirming that HFF significantly exerted influence on carbohydrate, lipid and energy metabolism of WAT as shown in our previous report on hepatic transcriptome (25). As expected, the high-dose MHP supplementation relieved this situation (**Figure 6K**) – nearly all involved pathways were redressed after MHP intake, suggesting that MHP could attenuate white adipose tissue transcriptome stress in HFF-diet-induced obese rats.

### 3.5 MHP facilitates obese rats weight loss partly through PPARγ signaling pathway

Storage of extra calories is an inborn ability of white adipose tissue. In the case of overnutrition, white adipose tissue usually experiences two scenarios, i.e., hyperplasia and hypertrophy. Adipocyte hyperplasia is commonly regarded as a healthy adaptation whereas adipocyte hypertrophy is associated with increased hypoxia which is regarded as an unhealthy state (43). In general, adipocyte hypertrophy is achieved in mature adipocytes via an increase in lipid accumulation or lipogenesis (i.e., triglyceride synthesis), which is synergetic with a decreased lipolysis (i.e., triglyceride breakdown). In contrast, adipocyte hyperplasia relies on a complicated adipogenesis process (44). The nuclear hormone receptor PPARγ is regarded as the master regulator of adipogenesis as it is indispensable for adipocyte differentiation. Mechanistically, TGF-β signals through SMAD3 and directly inhibits PPARγ-C/EBPα complex formation and, in consequence, adipogenesis (45).

**Figure 7:**
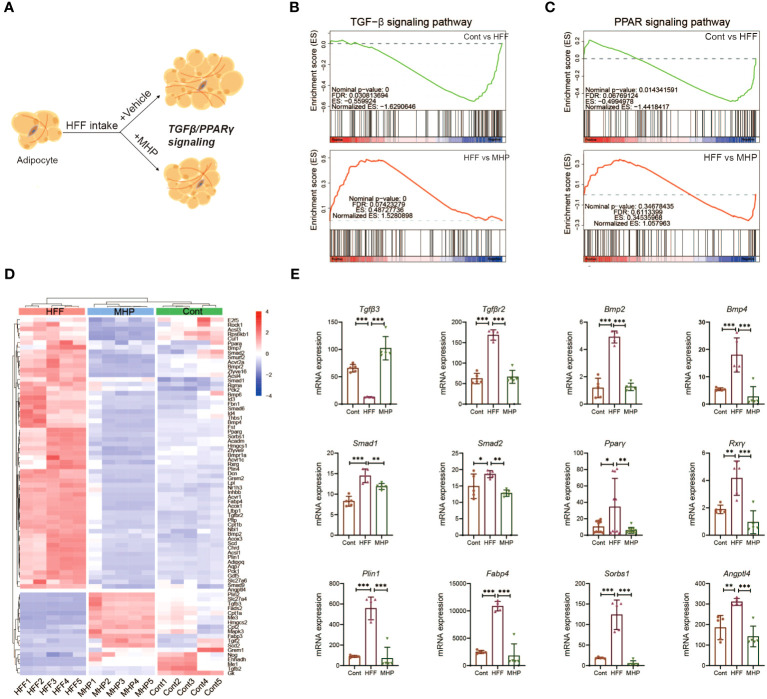
MHP promotes obese rats weight loss partly through TGF-β and PPARγ signaling pathways. **(A)** Schematic response of adipocytes to MHP intervention via TGF-β and PPARγ signaling pathways; **(B, C)** GSEA analysis of TGF-β and PPARγ signaling pathways; **(D)** Heatmap of the expressions of genes involved in TGF-β and PPARγ signaling pathway in iWAT; **(E)** mRNA expression level of typical genes. Data are expressed as mean ± SD (n=5). MHP, a mulberry and *Hippophae*-based solid beverage. GSEA, gene set enrichment analysis. iWAT, inguinal white adipose tissue. *p < 0.05, **p < 0.01, ***p < 0.001 vs. HFF group.

TGF-β and PPARγ signaling pathways play a crucial regulatory role in adipocyte hyperplasia and hypertrophy, which may be a key target for the treatment of obesity (**Figure 7A**). Take PPAR*γ* for instance, some of its ligands have been developed as clinically effective antidiabetic drugs (46, 47). Therefore, GSEA analysis on TGF-β signaling pathway and PPARγ signaling pathway in adipose tissue transcriptome were conducted. As compared to the control group, the HFF group exhibited a significant downregulation trend, which was reversed after MHP treatment (**Figures 7B, C**). The heatmap of genes related to adipocyte hyperplasia and hypertrophy reflected an obvious difference between the Cont and HFF groups, while, after MHP treatment, mRNA levels of differential genes tended to be normal (**Figure 7D**). As a transcription factor, PPARγ activates expression of various genes involved in fat deposition, such as *Lpl*, *Fatp1*, *CD36*, *Fabp4*, and *Plin*, by binding a functional peroxisome proliferator response element (PPRE) on their promoter in adipose tissue (48). In this study, MHP significantly suppressed the expression of *Pparγ* and its downstream target genes *Fabp4* and *Plin1* induced by HFF diet, suggesting that MHP may improve fat deposition by regulating the PPARγ pathway (**Figure 7E**).

In consideration of their structures and functions, the TGF-β superfamily ligands can be divided into two subfamilies: TGF-βs and BMPs (49). In our study, *Tgfβ3*, the key gene involved in adipocyte proliferation, exhibited a significant upregulation trend following MHP treatment (**Figure 7E**), indicating that MHP may improve the state of adipocyte hypertrophy by inhibiting TGFβ3-mediated adipocyte proliferation. Several genes involved in adipogenesis and lipid accumulation, for example, *Smad*1/2 and *Bmp*2/4, were robustly upregulated in the adipocytes under the HFF diet, but there was a significant inhibition of *Bmp*2/4 expression upon MHP intervention (**Figure 7E**). BMPs are pleiotropic proteins that regulate processes like cell-fate determination, proliferation, apoptosis and differentiation (50). BMP2 stimulates adipogenesis in 3T3-L1 cells by induction of PPARγ via mainly Smad-1/5/8 and p38 MAPK pathway (51). BMP4 promotes MSCs commitment to the adipocyte lineage and induces adipogenesis in a dose-dependent manner (52). Therefore, our findings suggest that MHP may exert its effects by modulating TGF-β and PPAR*γ* signaling pathways, thereby promoting adipocyte proliferation and inhibiting adipogenesis. Consequently, this could alleviate adipocyte hypertrophy and improve the unhealthy obese conditions.

### 3.6 Weight-loss effect of MHP is associated with its regulation on adipose fibrosis

**Figure 8:**
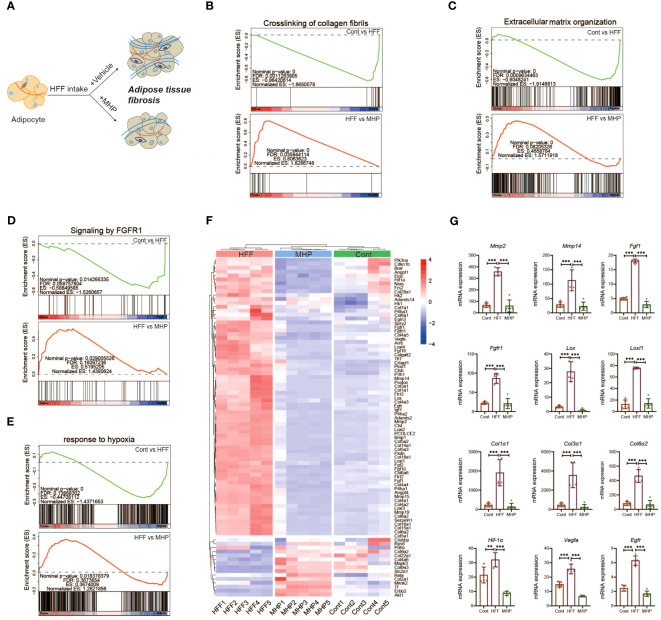
Efficacy of MHP on weight loss is associated with its regulation on FGFR1 pathway. **(A)** Schematic response of adipocytes to MHP intervention through improving adipose tissue dysfunction. GSEA analysis of pathways: crosslinking of collagen fibrils **(B)**, extracellular matrix organization **(C)**, signaling by FGFR1 **(D)** and response to hypoxia **(E)**. **(F)** Heatmap of the expressions of genes involved in aforementioned pathways in iWAT. **(G)** mRNA expression level of part of typical genes. MHP, a mulberry and *Hippophae*-based solid beverage. GSEA, gene set enrichment analysis. iWAT, inguinal white adipose tissue. Data are expressed as mean ± SD (n =5). **p < 0.01, ***p < 0.001 vs. HFF group.

Obesogenic adipogenesis is a pathological process which is closely related to ECM (extracellular matrix) (53). In physiological states, a balance between synthesis, deposit and degradation of ECM components exists. However, diet-induced obesity is usually accompanied with ongoing ECM remodeling, which likely results from an excess synthesis of fibrillar components, such as collagens I, III and VI, and an impaired degradation of these proteins (54). Excessive ECM deposition in adipose tissue is a hallmark of fibrosis, which is a major contributor to adipose tissue dysfunction (55). As described, Masson’s trichrome staining adipose tissue sections showed a higher content of collagen fiber in HFF-diet challenged rats as compared to the control group, but MHP reversed its progression (**Figure 6C**). Thus, we reasoned that the health benefit of MHP may also be due to its improvement on the adipose dysfunction (**Figure 8A**). In other words, MHP intervention may reduce ECM deposition as well as fibroblasts of adipocyte, consequently improving adipose tissue fibrosis challenged by HFF diet.

Since ECM remodeling is a complicated and dynamically changing process, we performed mRNA expression enrichment analysis of the gene set of extracellular matrix organization (**Figure 8C**). As expected, HFF diet changed the expression of ECM organization pathway genes whereas MHP intervention reversed this condition. As the matrix metalloproteinases (MMP) are regarded as marker proteins involved in adipose ECM remodeling, mRNA expression levels of MMP genes were also compared. As shown in **Figure 8G**, significant upregulation expression of MMP-2 and MMP-14 genes was found in HFF group rats, while it was normal in MHP group. It has been shown that MMP-2 is a key regulator of adipocyte differentiation, and high-expression of MMP-2 is necessary for adipocytes hyperplasia when responding to excess energy from HFF diet (56). Also, MMP14 is found dramatically upregulated in adipose tissue of obese mice induced by high-fat diet (57). Therefore, the efficacy of MHP is likely due to its protection against adipose tissue dysfunction.

Crosslinking of collagen, the main component of connective tissue surrounding adipocytes, is known to affect adipose remodeling, which is crucial for maintaining function and metabolic homeostasis of adipose tissue (58). In fact, increased interstitial fibrosis in WAT may lessen the flexibility of ECM and decrease the plasticity of adipose tissue as obesity progresses, which ultimately leads to adipose tissue dysfunction (55). Through GSEA analysis of the iWAT transcriptome, we found the trend back of crosslinking of collagen fibrils pathway after MHP intervention (**Figure 8B**). Similar results were also found in collagen formation pathway and assembly of collagen fibrils and other multimeric structures pathway (**Supplementary Figures S3A, B**).

In addition to ECM remodeling, adipose fibrosis also contributes greatly to adipose tissue dysfunction. We found that the signaling by FGFR1 pathway, which is associated with adipose tissue fibrosis, was markedly downregulated in the HFF group (**Figure 8D**), so did the downstream signaling of activated FGFR1 pathway (**Supplementary Figure S3C**). Interestingly, these two pathways were significantly upregulated after MHP treatment (**Figure 8D**). Further, the heatmap of the DEGs from signaling by FGFR1 pathway and crosslinking of collagen fibrils pathway suggests that MHP was able to improve HFF diet-induced obese adipose tissue fibrosis at the transcriptomic level (**Figure 8F**). As shown in **Figure 8G** and **Supplementary Figure S3D**, the critical genes involved in these two pathways in the HFF group, including *Fgf1, Fgfr1, Col1α1, Col1α3, Col6α2*, *Lox* and *Loxl1/2/3/4* showed a marked upregulation trend as compared with the control group, but showed a substantial downregulation trend after MHP intervention.

*Fgf1* is unique among the *Fgf* family in its ability to activate all four *Fgfrs* and their isoforms (59). It is reported that adipose *Fgf1* is dramatically induced in HFD-fed mice (60) and expression of *Fgf1* and *Fgfr1* in the adipose tissue of HFD-fed and ob/ob mice is significantly elevated when compared with normal diet-fed and lean control mice (61). In line with these reports, the expression of *Fgf1* and *Fgfr1* was significantly increased in the iWAT of HFF-induced obese rats but was normal after MHP treatment (**Figure 8G**). Collagen type I, III, and VI are highly expressed in adipose tissue (62) and diet induced obesity is found to promote the expressions of *Col1α1*, *Col3α1* and *Col6α3* in eWAT of C57/B6J mice (58). Moreover, *Col3α*, *Col6α*, and *Lox* gene expressions are downregulated by metformin and resveratrol, which are shown to decrease collagen deposition in adipose tissue induced by HFD (63). In fact, *Lox* (Lysyl oxidase), which catalyzes the formation of allysine and hydroxylysine, is crucial for collagen fiber crosslinking and thus for fibrosis development (64). Berberine is also found to suppress the expression of *Hif-1α* and *Lox* in eWAT at the state of obesity induced by HFD (65). Therefore, the efficacy of MHP is potentially associated with its protection against adipose tissue fibrosis.

As aforementioned, the adipocyte hypertrophy is usually along with an insufficient local blood supply and a subsequent locally hypoxic microenvironment in adipose tissue (66). Therefore, the genes related to hypoxia response may have some expression alterations in our case. For this, GSEA analysis of the iWAT transcriptome on the response to hypoxia pathway was carried out (**Figure 8E**). As expected, supplementation of MHP resulted in a strong downregulation trend for the key genes related to hypoxia, including *Hif-1α, Vegfr, Egfr* and *Igf1* (**Figure 8G**, **Supplementary Figure S3D**). Interestingly, an elevated expression of *Hif-1α, the* hypoxia-inducible factor-1 *α*, in obese adipose tissue is reported to trigger a potent pro-fibrotic transcriptional program, and inhibition or knockout *Hif-1α* can ameliorate the negative aspects of the obesity-associated fat pad expansion and adipose tissue fibrosis (66, 67).

Rapid tissue expansion during the development of obesity causes the neo-vasculature to struggle to keep up, and the mRNA expression of vascular endothelial growth factor (*Vegf*), a well-known *Hif-1α* target gene, is markedly elevated in HFD fed mice (55). On the other hand, hyperplastic expansion is preceded by angiogenesis but angiogenesis is often insufficient and occurs after hypertrophic adipocyte growth, which can result in hypoxia and tissue dysfunction (68, 69). Previous research has revealed that HFD increases expression of *Egfr* and its ligand amphiregulin in adipose tissue macrophages (ATMs). Furthermore*, Egfr* deletion in ATMs leads to an inhibition of resident ATM proliferation and monocyte infiltration into adipose tissue, which results in decreased obesity and insulin resistance (70). As described above, the expression of hypoxia-related genes was markedly increased in the HFF group, but it was normal after MHP intervention (**Figure 8G**), suggesting that the efficacy of MHP is related to its improvement on hypoxia condition of the hypertrophic adipocytes.

## 4 Conclusions

In the present study, body component analysis and histopathologic examination confirm that MHP can effectively facilitate weight loss and adiposity decrease in rat model of obesity. Pathway enrichment analysis with the DMs generated by serum metabolomic profiling and multivariant statistical analysis suggest that PPAR signal pathway was significantly altered when challenged by HFF diet while it was rectified after MHP intervention. Furthermore, the RNA-Seq based transcriptome data indicate that MHP intervention also rectified the alterations of white adipose tissue mRNA expressions in HFF-induced obese rats. Integrated omics reveals the efficacy of MHP against obesogenic adipogenesis was potentially with its regulation of PPARγ and FGFR1 signaling pathway. Collectively, our findings provide reliable evidence that MHP can improve obesity and an insight into the use of MHP in body weight management. Although full understanding of its mechanisms requires further work, MHP is a potential healthy product for body weight management.

## Data availability statement

The datasets presented in this study are included in the article/**Supplementary Materials**. The RNA-Seq data has been linked to the GenBank’s Sequence Read Archive here: https://www.ncbi.nlm.nih.gov/sra/PRJNA1063764. Further inquiries can be directed to the corresponding authors.

## Ethics statement

The animal study was approved by The Institutional Animal Care and Use Committee of Guangdong Pharmaceutical University. The study was conducted in accordance with the local legislation and institutional requirements.

## Author contributions

X-TZ: Writing – original draft, Data curation, Formal analysis. A-QZ: Data curation, Writing – original draft, Formal analysis. X-ML: Formal analysis, Resources, Writing – original draft. L-YS: Formal analysis, Data curation, Investigation, Writing – original draft. J-GY: Data curation, Investigation, Writing – original draft. NL: Data curation, Formal analysis, Investigation, Writing – original draft. S-SC: Formal analysis, Data curation, Investigation, Writing – original draft. ZH: Writing – review & editing, Conceptualization, Resources. X-LM: Conceptualization, Resources, Funding acquisition, Writing – original draft. K-PL: Writing – review & editing, Conceptualization, Data curation, Writing – original draft, Funding acquisition, Methodology.
