# Supplementary material for: Mulberry and Hippophae-based solid beverage promotes weight loss in rats by antagonizing white adipose tissue PPARγ and FGFR1 signaling
Source: Front Endocrinol (Lausanne). 2024 Mar 15;15:1344262. doi: 10.3389/fendo.2024.1344262 (PMC10978776; doi:10.3389/fendo.2024.1344262)
Supplement: Supplementary file 1 [file DataSheet_1.pdf]

Supplementary materials

Table S1 Test report on nutritional ingredients, heavy metals and microorganisms of MHP *

Item	Test results	Method
1-Deoxynojirimycin (DNJ), mg/100g	407.87	GB/T 40642-2021
Moisture, %	4.78	GB 5009.3-2016
Ash, %	5.7	GB 5009.4-2016
Lead, mg/kg	0.052	GB 5009.12-2017
Arsenic, mg/kg	0.27	GB 5009.11-2014
Mercury, mg/kg	<0.01	GB 5009.17-2021
Aerobic bacterial count, CFU/g	<10	GB 4789.2-2016
Mycete, CFU/g	<10	GB 4789.15-2016
Coliform bacteria, CFU/g	<10	GB 4789.3-2016
Staphylococcus aureus, CFU/25g	Not detected	GB 4789.10-2016
Salmonella, 0/25g	Not detected	GB 4789.4-2016
Energy, kJ/100g	1237	GB 28050-2011
Carbohydrate, g/100g	50.46	GB 28050-2011
Dietary fiber, g/100g	31.6	GB/T 22224-2008
Fat, g/100g	1.5	GB 5009.6-2016
Protein, g/100g	5.96	GB 5009.5-2016

*MHP, a commercial mulberry and *Hippophae*-based solid beverage, was produced in a pilot plant that adhered to the strict guidelines of Good Manufacturing Practice. The nutritional ingredients, heavy metals and microorganisms were tested following a serial of national standards of China for food safety and quality control as indicated.

Table S2 Differential metabolites generated by serum metabolomics analysis of Cont vs HFF*

NO.	Metabolites	*m/z*	RT(s)	FC	*p*-value	VIP value	Molecular Formula	KEGG ID
1	2-Hydroxybutyric acid	104.0534	72.2	1.42	0.008	1.578	$C_4H_8O_3$	C05984
2	Benzaldehyde	107.0496	100.6	3.98	0.000	1.985	C_7H_6O	C00261
3	m-Cresol	109.1016	287.6	0.85	0.018	1.401	C_7H_8O	C01467
4	Creatinine	114.0665	49.9	0.46	0.000	2.143	$C_4H_7N_3O$	C00791
5	L-2,4-diaminobutyric acid	118.0656	187.1	0.74	0.001	1.883	$C_4H_{10}N_2O_2$	C03283
6	5-Hydroxypentanoic acid	119.0734	187.1	0.66	0.048	1.233	$C_5H_{10}O_3$	C02804
7	L-Threonine	120.0660	92.0	0.83	0.012	1.494	$C_4H_9NO_3$	C00188
8	Niacinamide	123.0558	72.2	0.40	0.024	1.442	$C_6H_6N_2O$	C00153
9	Nicotinic acid	124.0863	687.8	0.71	0.000	1.953	$C_6H_5NO_2$	C00253
10	Isoquinoline	130.0656	296.7	0.16	0.000	1.986	C_9H_7N	C06323
11	Pipecolic acid	130.0505	72.2	1.32	0.035	1.303	$C_6H_{11}NO_2$	C00408
12	Glutaric acid	131.9749	35.5	7.60	0.000	2.096	$C_5H_8O_4$	C00489
13	Isophorone	139.1124	391.7	0.75	0.000	1.965	$C_9H_{14}O$	C14743
14	Phosphonoacetate	140.0709	109.9	1.21	0.002	1.829	$C_2H_5O_5P$	C05682
15	(S)-2-Methylmalate	149.0603	100.7	0.87	0.020	1.390	$C_5H_8O_5$	C02614
16	L-2-Hydroxyglutaric acid	148.9760	36.2	0.70	0.003	1.708	$C_5H_8O_5$	C03196
17	L-Histidine	156.0775	43.8	0.50	0.011	1.537	$C_6H_9N_3O_2$	C00135
18	Uracil 5-carboxylate	156.9653	34.4	0.36	0.020	1.464	$C_5H_4N_2O_4$	C03030
19	4,5-Dihydroorotic acid	158.9643	240.0	0.59	0.021	1.433	$C_5H_6N_2O_4$	C00337
20	L-Rhamnono-1,4-lactone	162.0592	82.5	5.03	0.001	2.000	$C_6H_{10}O_5$	C02991
21	L-Phenylalanine	166.0871	285.7	0.86	0.013	1.471	$C_9H_{11}NO_2$	C00079
22	D-synephrine	168.0918	100.7	0.81	0.003	1.676	$C_9H_{13}NO_2$	C01869
23	4-Quinolinecarboxylic acid	172.9570	61.6	6.18	0.000	2.071	$C_{10}H_7NO_2$	C06414
24	N-Acetylornithine	175.1085	76.0	1.66	0.010	1.565	$C_7H_{14}N_2O_3$	C00437
25	Sorbitol	182.9858	424.2	0.50	0.037	1.324	$C_6H_{14}O_6$	C00794
26	3,4-Dihydroxymandelic acid	184.9845	36.7	1.15	0.010	1.556	$C_8H_8O_5$	C05580
27	2-Keto-6-acetamidocaproate	188.0908	296.5	0.66	0.007	1.607	$C_8H_{13}NO_4$	C05548
28	N-Acetyl-L-phenylalanine	208.0979	293.3	1.18	0.013	1.557	$C_{11}H_{13}NO_3$	C03519
29	L-Arogenate	210.0773	186.2	1.18	0.005	1.701	$C_{10}H_{13}NO_5$	C00826
30	Porphobilinogen	226.1811	646.7	1.69	0.000	1.939	$C_{10}H_{14}N_2O_4$	C00931
31	Anserine	241.1306	42.9	1.52	0.022	1.543	$C_{10}H_{16}N_4O_3$	C01262
32	17α-Estradiol	272.1860	249.4	1.18	0.036	1.346	$C_{18}H_{24}O_2$	C02537
33	4-Hydroxycinnamoylagmatine	276.1440	72.5	0.49	0.017	1.518	$C_{14}H_{20}N_4O_2$	C04498
34	α-dimorphecolic acid	279.2329	515.7	2.38	0.000	1.970	$C_{18}H_{32}O_3$	C14767
35	Oleic acid	282.2784	685.4	7.22	0.000	2.102	$C_{18}H_{34}O_2$	C00712
36	13-L-Hydroperoxylinoleic acid	295.2278	505.7	0.57	0.037	1.363	$C_{18}H_{32}O_4$	C04717

37	5'-Methylthioadenosine	298.0972	164.7	4.34	0.000	2.166	$C_{11}H_{15}N_5O_3S$	C00170
38	Tridemorph	298.3110	648.6	18.47	0.000	2.297	$C_{19}H_{39}NO$	C11285
39	Palmitoylethanolamide	300.2899	589.4	0.75	0.036	1.310	$C_{18}H_{37}NO_2$	C16512
40	2,3-Dinor-8-iso PGF2α	309.2060	524.3	0.10	0.035	1.416	$C_{18}H_{30}O_5$	C14794
41	N,N-Dimethylsphing-4-enine	310.3107	535.7	0.85	0.020	1.421	$C_{20}H_{41}NO_2$	C13914
42	Decanoyl-L-carnitine	316.2492	384.1	2.36	0.006	1.611	$C_{17}H_{33}NO_4$	C03299
43	Desmosterol	384.3473	443.0	1.85	0.000	1.929	$C_{27}H_{44}O$	C01802
44	Adenosine 5'-phosphate disodium	391.2847	573.2	2.88	0.006	1.778	$C_{10}H_{12}N_5O_7P_2Na$	-
45	Allocholic acid	408.3699	650.4	3.77	0.049	1.218	$C_{24}H_{40}O_5$	C00695
46	Gitogenin	432.3196	520.8	3.23	0.040	1.304	$C_{27}H_{44}O_4$	C08899
47	L-Olivosyl-oleandolide	516.3046	474.9	0.37	0.006	1.652	$C_{26}H_{44}O_{10}$	C11991
48	Fucoxanthin	659.4285	621.3	0.01	0.033	1.416	$C_{42}H_{58}O_6$	C08596
49	L-Erythrulose	119.0356	49.4	0.39	0.003	1.829	$C_4H_8O_4$	C02045
50	Ketoleucine	129.0563	97.7	0.66	0.000	2.139	$C_6H_{10}O_3$	C00233
51	L-Malic acid	133.0148	91.4	0.65	0.001	1.850	$C_4H_6O_5$	C00149
52	Phenyl acetate	134.8953	60.5	0.66	0.001	1.797	$C_8H_8O_2$	C00548
53	D-Xylose	149.0100	545.9	1.35	0.010	1.582	$C_5H_{10}O_5$	C00181
54	D-Phenylalanine	165.0200	91.7	2.08	0.007	1.585	$C_9H_{11}NO_2$	C02265
55	L-Arginine	173.1059	76.3	0.35	0.008	1.561	$C_6H_{14}N_4O_2$	C00062
56	Guanidinosuccinic acid	174.9542	237.4	2.02	0.020	1.386	$C_5H_9N_3O_4$	C03139
57	2,4-Dinitrophenol	183.0055	253.4	1.67	0.011	1.524	$C_6H_4N_2O_5$	C02496
58	Equol	242.1849	422.0	2.49	0.000	2.016	$C_{15}H_{14}O_3$	C14131
59	Uridine	243.0628	71.9	50.72	0.000	2.384	$C_9H_{12}N_2O_6$	C00299
60	16-Hydroxy hexadecanoic acid	271.2292	503.7	1.31	0.009	1.526	$C_{16}H_{32}O_3$	C18218
61	Dibutyl phthalate	277.1453	376.0	1.28	0.034	1.300	$C_{16}H_{22}O_4$	C14214
62	(9E)-Octadecenoic acid	282.2554	550.6	1.29	0.013	1.479	$C_{18}H_{34}O_2$	C01712
63	2-Methoxyestradiol	283.1712	549.8	1.20	0.038	1.279	$C_{19}H_{26}O_3$	C05302
64	9-OxoODE	293.2128	516.0	1.59	0.002	1.757	$C_{18}H_{30}O_3$	C14766
65	13S-hydroxyoctadecadienoic acid	295.2287	470.3	2.69	0.000	2.231	$C_{18}H_{32}O_3$	C14762
66	Arachidic acid	311.2968	673.6	7.74	0.000	2.363	$C_{20}H_{40}O_2$	C06425
67	11,12-EET	319.2285	507.1	0.48	0.001	1.844	$C_{20}H_{32}O_3$	C14770
68	20-HETE	319.2290	537.3	1.47	0.040	1.268	$C_{20}H_{32}O_3$	C14748
69	11,12,15-THETA	335.2236	363.8	1.26	0.033	1.363	$C_{20}H_{34}O_5$	C14782
70	Corticosterone	345.2079	388.5	1.27	0.013	1.476	$C_{21}H_{30}O_4$	C02140
71	Deoxycorticosterone acetate	371.0669	535.8	0.59	0.007	1.558	$C_{23}H_{32}O_4$	C14554
72	LysoPA(16_0_0_0)	409.2370	466.1	0.44	0.000	1.878	$C_{19}H_{39}O_7P$	C04036
73	α-Tocotrienol	423.3265	689.6	0.06	0.000	2.130	$C_{29}H_{44}O_2$	C14153

*Taking VIP value > 1 and p value < 0.05 as the thresholds. *m/z*, mass to charge ratio. RT(s), retention

time(second). FC (Fold change) was calculated as the logarithm of the average mass response (area) ratio between the two classes (i.e., Fold change = log_2[HFF/Cont]). Cont, control group; HFF, a high-fat high-fructose diet fed group (n=8, each group).

Table S3 Differential metabolites generated by serum metabolomics analysis of HFF vs MHP

NO.	Metabolites	*m/z*	RT(s)	FC	*p*-value	VIP value	Molecular formula	KEGG ID
1	Benzaldehyde	107.0496	100.6	0.35	0.000	1.581	C_7H_6O	C00261
2	Dihydrouracil	115.0395	85.3	1.69	0.000	1.463	$C_4H_6N_2O_2$	C00429
3	L-Proline	116.0711	72.5	2.03	0.019	1.029	$C_5H_9NO_2$	C00148
4	Styrene Oxide	120.0449	117.2	0.59	0.039	1.018	C_8H_8O	C02083
5	Glutaric acid	131.9749	35.5	0.05	0.000	1.668	$C_5H_8O_4$	C00489
6	L-Leucine	132.1026	75.1	1.14	0.034	1.045	$C_6H_{13}NO_2$	C00123
7	6-Hydroxyhexanoic acid	133.0865	102.6	12.82	0.000	1.622	$C_6H_{12}O_3$	C06103
8	Ornithine	133.0976	43.2	4.68	0.006	1.322	$C_5H_{12}N_2O_2$	C01602
9	Perillyl alcohol	135.1174	470.0	1.18	0.024	1.100	$C_{10}H_{16}O$	C02452
10	Isophorone	139.1124	391.7	1.31	0.001	1.342	$C_9H_{14}O$	C14743
11	4-Hydroxycinnamic acid	146.9805	688.7	1.78	0.000	1.638	$C_9H_8O_3$	C00811
12	(S)-2-Methylmalate	149.0603	100.7	1.13	0.038	1.055	$C_5H_8O_5$	C02614
13	Uracil 5-carboxylate	156.9653	34.4	0.53	0.034	1.022	$C_5H_4N_2O_4$	C03030
14	L-Rhamnono-1,4-lactone	162.0592	82.5	0.20	0.001	1.356	$C_6H_{10}O_5$	C02991
15	L-Phenylalanine	166.0871	285.7	1.13	0.040	1.041	$C_9H_{11}NO_2$	C00079
16	D-synephrine	168.0918	100.7	1.20	0.006	1.276	$C_9H_{13}NO_2$	C01869
17	Phosphoserine	167.9967	384.7	1.68	0.015	1.138	$C_3H_8NO_6P$	C01005
18	4-Quinolinecarboxylic acid	172.9570	61.6	0.06	0.000	1.698	$C_{10}H_7NO_2$	C06414
19	3-Isopropylmalate	176.0715	357.3	0.66	0.039	1.019	$C_7H_{12}O_5$	C04411
20	Ureidosuccinic acid	176.0412	50.2	2.18	0.027	1.042	$C_5H_8N_2O_5$	C00438
21	Serotonin	177.1031	83.9	13.19	0.000	1.479	$C_{10}H_{12}N_2O$	C00780
22	3,4-Dihydroxymandelic acid	184.9845	36.7	1.27	0.001	1.363	$C_8H_8O_5$	C05580
23	Dodecanedioic acid	213.1493	385.2	0.26	0.004	1.119	$C_{12}H_{22}O_4$	C02678
24	Dethiobiotin	215.1396	77.2	0.54	0.028	1.123	$C_{10}H_{18}N_2O_3$	C01909
25	N-a-Acetylcitrulline	217.1079	309.9	1.44	0.006	1.111	$C_8H_{15}N_3O_4$	C15532
26	Hydroxykynurenine	224.1288	264.5	0.78	0.005	1.269	$C_{10}H_{12}N_2O_4$	C02794
27	Xanthoxic acid	267.1603	365.2	19.82	0.001	1.377	$C_{15}H_{22}O_4$	C13454
28	Stearic acid	267.1724	517.2	0.41	0.015	1.185	$C_{18}H_{36}O_2$	C01530
29	Xanthosine	285.0823	72.4	50.61	0.001	1.502	$C_{10}H_{12}N_4O_6$	C01762
30	8-HETE	303.2322	553.1	0.91	0.006	1.185	$C_{20}H_{32}O_3$	C14776
31	(-)-Epigallocatechin	307.0829	53.3	2.06	0.001	1.386	$C_{15}H_{14}O_7$	C12136
32	2,3-Dinor-8-iso PGF2α	309.2060	524.3	0.59	0.026	1.097	$C_{18}H_{30}O_5$	C14794
33	Aflatoxin B1	312.3633	533.2	0.63	0.005	1.298	$C_{17}H_{12}O_6$	C06800
34	Spectinomycin	333.1566	263.1	4.73	0.002	1.368	$C_{14}H_{24}N_2O_7$	C02078

35	12-Keto-tetrahydro-leukotriene B4	336.3110	591.4	0.73	0.020	1.044	$C_{20}H_{32}O_4$	C02165
36	Sucrose	343.2989	406.0	0.74	0.000	1.488	$C_{12}H_{22}O_{11}$	C00089
37	Aldosterone	361.2228	546.9	23.69	0.000	1.536	$C_{21}H_{28}O_5$	C01780
38	Desmosterol	384.3473	443.0	0.69	0.020	1.075	$C_{27}H_{44}O$	C01802
39	3α,7α-Dihydroxy-12-oxo-5β-cholanate	389.2597	249.6	38.81	0.000	1.474	$C_{24}H_{38}O_5$	C01292
40	β-Sitosterol	397.3837	614.1	93.29	0.000	1.560	$C_{29}H_{50}O$	C01753
41	7α,12α-Dihydroxy-5β-cholestan-3-one	401.3431	564.9	37.52	0.000	1.549	$C_{27}H_{46}O_3$	C05453
42	Nobiletin	403.1392	435.0	0.27	0.013	1.170	$C_{21}H_{22}O_8$	C10112
43	Allocholic acid	408.3699	650.4	5.56	0.006	1.142	$C_{24}H_{40}O_5$	C00695
44	Hecogenin	413.3062	596.6	0.19	0.028	1.108	$C_{27}H_{42}O_4$	C08902
45	14α-Hydroxy-5β-cholest-7-ene-3,6-dione	414.3043	436.0	30.53	0.019	1.108	$C_{27}H_{42}O_3$	C16509
46	Sodium deoxycholate	415.2118	476.1	42.6	0.000	1.586	$C_{24}H_{39}O_4$. Na	C11171
47	Mifepristone	429.2614	399.3	44.45	0.003	1.428	$C_{29}H_{35}NO_2$	C07652
48	2,22-Dideoxy-3-dehydroecdysone	430.2950	382.6	8.71	0.001	1.449	$C_{27}H_{42}O_4$	C16498
	3β,5β-Ketotriol	432.3128	383.3	70.47	0.000	1.644	$C_{27}H_{44}O_4$	C16494
50	3-Dehydro-2-deoxyecdysone	446.2948	358.4	2.93	0.024	1.014	$C_{27}H_{42}O_5$	C16497
51	3-Dehydroecdysone	462.2855	373.2	19.26	0.000	1.597	$C_{27}H_{42}O_6$	C02513
52	Antibiotic JI-20A	482.2875	449.8	11.57	0.001	1.461	$C_{19}H_{39}N_5O_9$	C17704
53	Antibiotic G-418	497.2896	284.6	17.21	0.000	1.574	$C_{20}H_{40}N_4O_{10}$	C17703
54	Taurocholic acid	498.2896	353.4	187.61	0.000	1.704	$C_{26}H_{45}NO_7S$	C05122
55	β-Carotene	536.1745	671.7	2.56	0.011	1.089	$C_{40}H_{56}$	C02094
56	Rutin	610.1815	660.9	49.73	0.001	1.419	$C_{27}H_{30}O_{16}$	C05625
57	Fucoxanthin	659.4285	621.3	0.32	0.010	1.213	$C_{42}H_{58}O_6$	C08596
58	Thioetheramide PC	735.5653	581.1	0.31	0.015	1.137	$C_{40}H_{84}N_2O_5PS$	C04873
59	Fumaric acid	116.0357	45.3	1.26	0.004	1.789	$C_4H_4O_4$	C00122
60	Pyroglutamic acid	128.0353	46.2	0.60	0.008	1.639	$C_5H_7NO_3$	C01879
61	3-Methyl-2-oxovaleric acid	129.0561	108.2	0.85	0.012	1.534	$C_6H_{10}O_3$	C03465
62	trans-Cinnamate	147.0458	91.0	0.80	0.024	1.395	$C_9H_8O_2$	C00423
63	Phthalic acid	165.0416	666.0	1.54	0.028	1.461	$C_8H_6O_4$	C01606
64	Uric acid	167.0218	48.3	0.22	0.001	2.023	$C_5H_4N_4O_3$	C00366
65	Methyl β-D-galactoside	194.0830	467.7	2.70	0.005	1.654	$C_7H_{14}O_6$	C03619
66	N-Acetyl-D-glucosamine	221.1552	519.6	0.60	0.047	1.383	$C_8H_{15}NO_6$	C00140
67	N2-Malonyl-D-tryptophan	271.0711	134.8	0.41	0.006	1.714	$C_{14}H_{14}N_2O_5$	C03414
68	Glycitein	284.0717	381.9	0.64	0.005	1.765	$C_{16}H_{12}O_5$	C14536
69	9,10-Epoxyoctadecenoic acid	295.2287	490.6	0.83	0.041	1.331	$C_{18}H_{32}O_3$	C14825
70	Arachidic acid	311.2968	673.6	0.38	0.001	1.915	$C_{20}H_{40}O_2$	C06425
71	12-KETE	317.2127	527.3	0.68	0.008	1.664	$C_{20}H_{30}O_3$	C14807
72	Adrenic acid	331.2653	644.1	2.46	0.002	1.828	$C_{22}H_{36}O_2$	C16527

73	Behenic acid	339.3280	589.0	0.49	0.025	1.427	$C_{22}H_{44}O_2$	C08281
74	Troxilin B3	353.2337	379.8	2.82	0.015	1.519	$C_{20}H_{34}O_5$	C14811
75	Deoxycorticosterone acetate	371.0669	535.8	1.48	0.014	1.604	$C_{23}H_{32}O_4$	C14554
76	Glycocholic acid	464.3008	329.2	0.31	0.000	2.005	$C_{26}H_{43}NO_6$	C01921

*Taking VIP value > 1 and p value < 0.05 as the thresholds. *m/z*, mass to charge ratio. RT(s), retention time (second). FC (Fold change) was calculated as the logarithm of the average mass response (area) ratio between the two classes (i.e., Fold change = $\log_2$[HFF/MHP]). HFF, a high-fat high-fructose diet fed group; MHP, a high-dose of MHP treated group (n=8, each group). MHP, a commercial mulberry and *Hippophae*-based solid beverage.

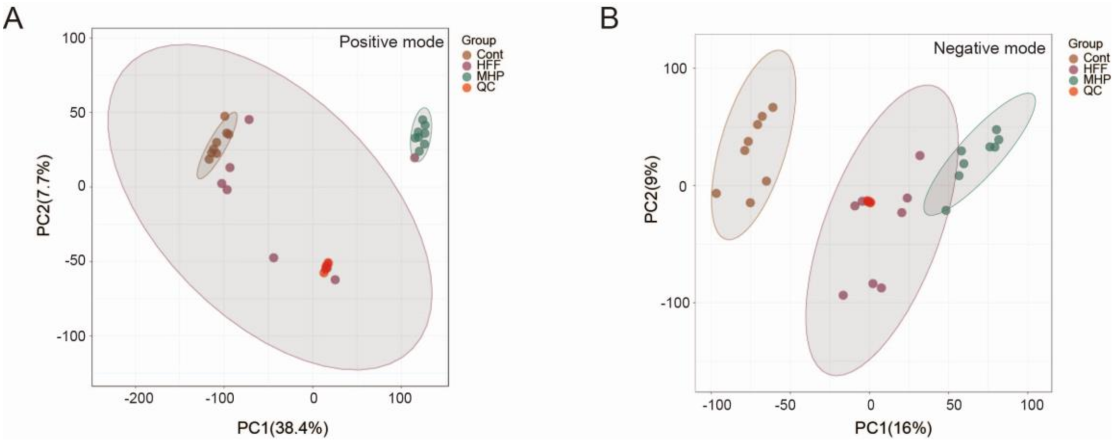

Figure S1 Quality control analysis of the LC-MS data acquired under positive and negative ion mode. Untargeted metabolic analysis was performed through Vanquish UHPLC tandem Orbitrap Exploris120 MS system. A small aliquot of each sample was pooled together as the quality control sample. Principal component analysis (PCA) was applied to discriminate the groups by R ropls (v1.22.0) package. (A-B) PCA score plots between every two groups. Cont, control group; HFF, a high-fat high-fructose diet fed group; MHP, a high dose of MHP supplemented group (n=8, each group). QC, quality control. MHP, a commercial mulberry and *Hippophae*-solid beverage.

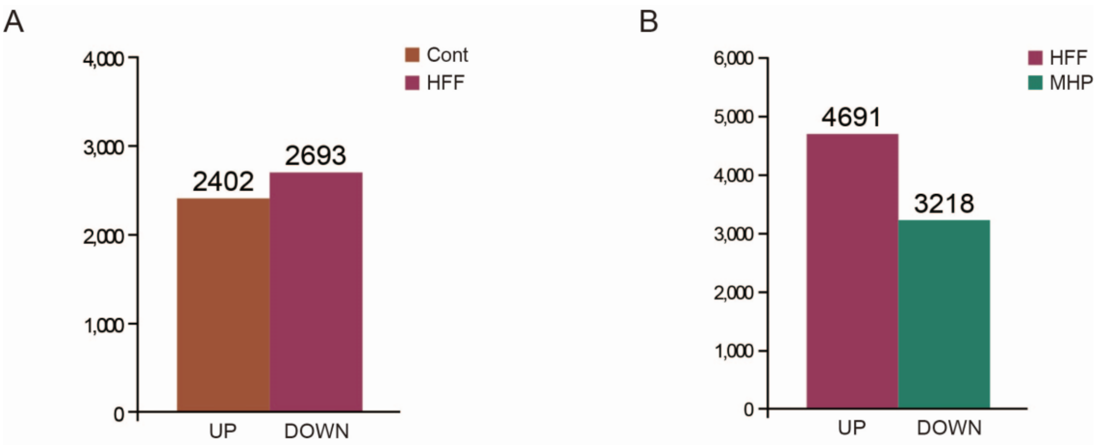

Figure S2 Differentially expressed genes generated from iWAT transcriptomics analysis. mRNAs differential expression analysis was performed through DESeq2 software between two different groups. Genes with p value < 0.05 and |Fold change|> 2 were categorized as differentially expressed genes (DEGs). (A) Statistical result of DEGs between Cont and HFF group. (B) Statistical result of DEGs between HFF and MHP group. iWAT, inguinal white adipose tissue. Cont, control group. HFF, a high-fat high-fructose diet fed group. MHP, a high-dose of MHP supplemented group (n=5, each group). MHP, a commercial mulberry and *Hippophae*-solid beverage.

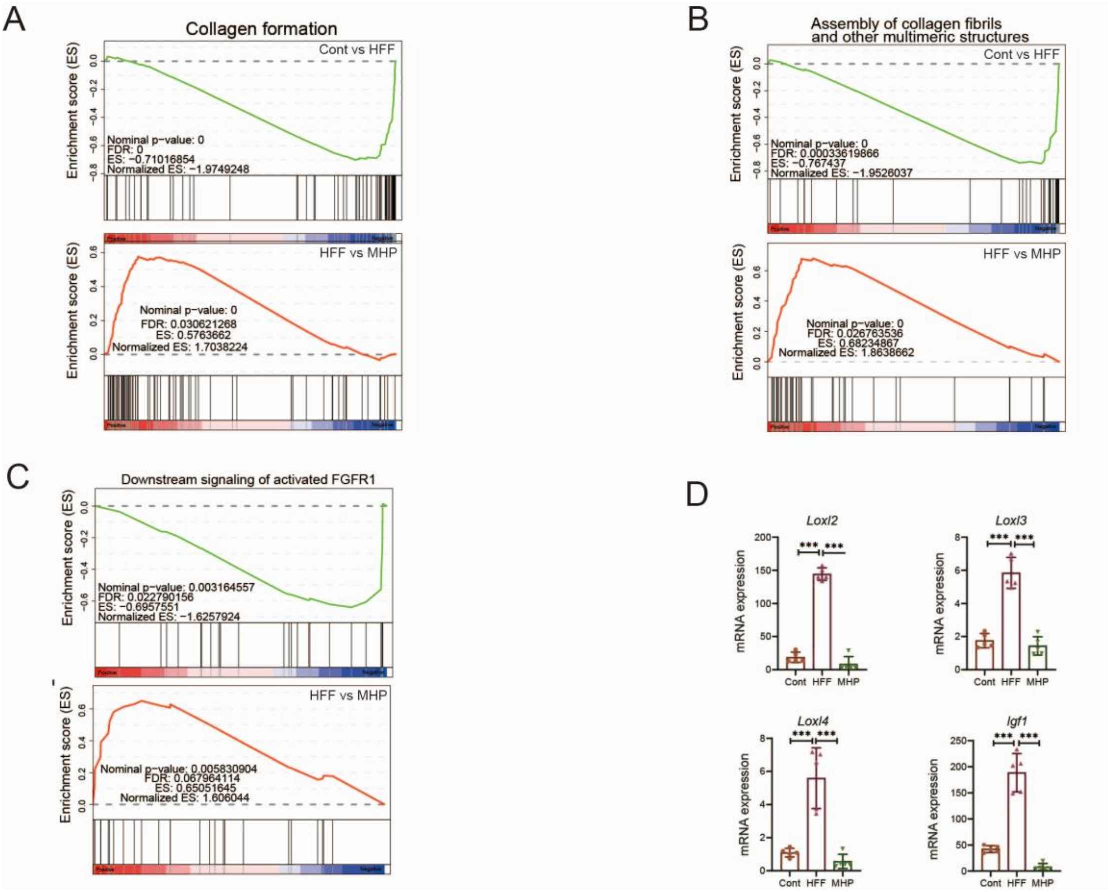

Figure S3 Effects of MHP on mRNA expression of some pathways related to adipose dysfunction in iWAT. (A-C) GSEA analysis of signaling by (1) collagen formation, (2) assembly of collagen fibrils and other multimeric structures and (3) downstream signaling of activated FGFR1. (D) mRNA expression level of part of typical genes. Cont, control group. HFF, a high-fat high-fructose diet fed group. MHP, a high dose-MHP supplemented group. MHP, a mulberry and *Hippophae*-based solid beverage. GSEA, gene set enrichment analysis. iWAT, inguinal white adipose tissue. Data are expressed as mean ± SD (n =5). $^{**}p < 0.01$, $^{***}p < 0.001$ vs. HFF group.
